# Factors associated with intention to breastfeed in Vietnamese mothers: A cross-sectional study

**DOI:** 10.1371/journal.pone.0279691

**Published:** 2023-12-12

**Authors:** Duong Thi Thuy Doan, Colin Binns, Andy Lee, Yun Zhao, Minh Ngoc Pham, Hoa Thi Phuong Dinh, Chuong Canh Nguyen, Ha Thi Thu Bui

**Affiliations:** 1 Hanoi University of Public Health, Hanoi, Vietnam; 2 School of Public Health, Curtin University, Western Australia, Australia; 3 Thai Nguyen University of Medicine and Pharmacy, Thai Nguyen, Vietnam; 4 Hanoi Gynecology and Obstetrics Hospital, Hanoi, Vietnam; Medical Research Council, SOUTH AFRICA

## Abstract

**Introduction:**

Breastfeeding has many benefits for mothers, children, and the environment over both the short and longr-term. Prenatal intention to breastfeed is a powerful predictor of short-term breastfeeding outcomes.

**Objective:**

This study aims to analyze breastfeeding intentions, including the intention to feed infants with breastmilk only and to continue exclusive breastfeeding to 6 months among pregnant mothers in Hanoi, Vietnam.

**Methods:**

The analysis included 1230 singleton mothers, between 24- and 36-weeks’ gestation, who attended antenatal clinics in two hospitals in Hanoi in 2020.

**Results:**

The proportion of mothers with an “breastfeeding intention” (i.e., intention to feed an infant with breastmilk only) and “exclusive breastfeeding intention” to 6 months was 59.9% and 41.7%, respectively. Mothers who were 25 years or older (aOR = 1.35, 95%CI:1.00–1.81), had an undergraduate educational degree or higher (aOR = 1.38, 95%CI: 1.08–1.76), had observed another woman breastfeeding (aOR = 1.43, 95%CI: 1.03–2.00), were not living with parents-in-law (aOR = 1.34, CI: 1.05–1.70), and were multiparous (aOR = 1.60, 95%CI: 1.16–2.19) had higher odds of “exclusive breastfeeding intention” to 6 months. Among primiparous women, those who thought their husbands support breastfeeding were more likely to intend to feed an infant with breastmilk only. Among multiparous women, feeding the previous child with breastmilk exclusively before the introduction of complementary foods and not giving solid foods together with water until 6 months, were significant predictors for both breastfeeding intentions.

**Conclusion:**

Mothers without exclusive breastfeeding experience should be provided with greater support to promote exclusive breastfeeding intention and outcomes.

## Introduction

The benefits of breastfeeding for both infants and mothers are numerous and substantial, across the short- and long- term. Breastfeeding protects infants against many infections, including diarrheal disease and respiratory tract infections [1,2], and creates a healthy microbiome that is associated with lower rates of chronic illness later in life [3–6]. Increasing the exclusive breastfeeding rate among children under 6 months and continued breastfeeding for those aged 6‐23 months could potentially save 823,000 children’s lives annually [4]. Benefits for mothers include the reduction of complications and depression during post-partum periods [7–9], and are also associated with lower rates of breast and ovarian cancer, hypertension, and diabetes later years in life [10–12].

Prenatal intention to breastfeed is a powerful predictor of short-term breastfeeding outcomes including initiated breastfeeding, exclusive breastfeeding, or any breastfeeding for both the first and second child [13–17]. Having the intention to breastfeed for six months or more was associated with feeding any breastmilk [14]. For those who initiated breastfeeding, one of the strongest predictors of discontinuing breastfeeding was low intention to breastfeed [16]. Mothers who intended to breastfeed had more knowledge about potential food contaminants and consulted more sources of information about nutrition and diet than mothers who did not intend to breastfeed [18].

Recent studies have shown that many factors can affect breastfeeding intentions. These factors include parity, previous breastfeeding experience [15,19–21], seeing other women breastfeed [22,23], having a supportive partner [19,20,22,24,25], not living with any family-in-law [20], breastfeeding knowledge [26–28], and having a positive attitude and beliefs regarding the benefits of breastfeeding [20,22]. Each of these are modifiable factors that health practitioners can target to improve breastfeeding rates. In addition, demographic variables including the place of residence [27], occupation [26], household economic status [20,26], maternal age [20,27], and maternal education [19,20,27] are also associated with breastfeeding intention.

Vietnam is a middle-income country in Southeast Asia with a population of 99 million. While an increase in the breastfeeding rate has been observed in some other developing countries [29], it continues to decline in Vietnam, particularly in urban areas [30,31]. In Vietnam, the breastfeeding rate within one hour of birth was only 45.2%, while the exclusive breastfeeding rate within the three first days after birth was even lower at 25.8% in 2012. The rate of exclusive breastfeeding through the first 6 months (24-hour recall) was 12.6%, lower than the national level (24%) [32]. These rates indicate a very low coverage and at present cannot be achieving the Vietnam’s 2030 targets [33]. Further, recent years have shown more intensive advertising of infant formula, and breastmilk substitutes [34]. The recent COVID-19 pandemic and the increased promotion of breastmilk substitutes on social media are influencing mothers’ infant feeding practices [35]. Thus, studies of the intention to breastfeed of Vietnamese mothers have become increasingly essential in light of rising promotion and with the resulting declines in rates of exclusive breastfeeding.

In this paper, we surveyed and analyzed factors associated with breastfeeding intentions among pregnant mothers in Hanoi, Vietnam.

## Materials and methods

### Study design

Data were collected in a cross-sectional survey and analyse. This survey was the baseline of a randomized controlled trial for which the protocol has been previously reported [36]. Data were collected from May to September 2020 from Dong Anh general district hospital and Hanoi Gynecology and Obstetrics hospital in Hanoi, Vietnam. Mothers were consecutively recruited from the two hospitals until the required sample size was reached.

### Study participants

All pregnant women who attended antenatal clinics in the two hospitals were approached by research assistants with a letter of invitation and information about the study. Women were eligible to participate if they were between 24 and 36 weeks of pregnancy, and excluded if they were referred by other hospitals for high-risk pregnancy treatments or received advice from doctors against breastfeeding because of their health condition [37]. Once eligibility was established and written consent was obtained, women then were asked to complete the initial data collection form. Out of 1691 mothers who were approached, 1387 mothers (82%) agreed to participate in the survey and 1230 mothers (response rate: 72.7%) completed the questions on breastfeeding intentions and previous experience with breastfeeding. Our sample size of 1230 mothers satisfied the required sample size under simple random sampling for a cross-sectional study [38], using the formula given below [38]:

$$n=\frac{z_{1-\alpha /2}^{2}p(1-p)}{d^{2}},$$

where *n* is the required sample size, *z*_(1-a/2)_ is the critical z score (= 1.96 for a 95% confidence level), p is the assumed true proportion (assumed 50% of mothers who have breastfeeding intention to estimate the largest conservative required sample size), and d is the margin of error (assumed 0.03 as the desired precision). This sample size was also sufficient to perform a multivariable logistic regression analysis with 9 covariates [39].

### Data collection and key measures

Face-to-face interviews were conducted by eight trained research assistants at the two recruiting hospitals. The interviewers were health workers who were trained and monitored by researchers regularly to ensure that interview protocols were strictly followed. Data were collected and managed using REDCap [40]. Each interview lasted 20 to 30 minutes and included a range of questions (see Supplement files 5 and 6 for the questionnaire in Vietnamese and in English, respectively). The questions on breastfeeding were adapted from Multiple Indicator Cluster Surveys 4, Questionnaire for Individual Women [41], and previous studies on breastfeeding which had been implemented in Vietnam [42–44]. Other questions on independent factors associated with breastfeeding were developed from a literature review. The questionnaire was developed by breastfeeding experts from both Vietnam and Australia in English and then translated into Vietnamese. It was pilot-tested with 20 pregnant women, 10 per hospital, and revised to ensure that questions were correctly interpreted in the local cultural context. The questionnaire was then back-translated into English to optimize the accuracy by a second independent translator and verified by the lead investigator before being buit in REDCap. Following creation in REDCap, the questionnaire was tested again among the research team and with 10 mothers to make sure intricate skip patterns and logic were employed seamlessly. Variables were included in the analysis after reviewing previous studies on breastfeeding intention as listed below.

#### Dependent variables

We used two dependent (outcome) variables in this study: “breastfeeding intention” and “exclusive breastfeeding intention” to 6 months. To assess “breastfeeding intention”–mothers were asked, “What method do you plan to use to feed your newborn for the first six months?” Response options included: breastfeeding only, formula feeding, both breast, and formula feeding, or don’t know yet. The variable “breastfeeding intention” is coded 1 if mothers answered “breastfeeding only” and as 0 for other options.

To assess “intention to breastfeed exclusively” for 6 months, in addition to the above question, mothers were asked two more questions: “when will you plan to feed her newborn with complementary foods?” and “when will you plans to feed her newborn with water or liquid?”. Those who planned not to give complementary foods and water until six months of birth and intended to breastfeed were defined as having the intention to breastfeed exclusively for 6 months. The corresponding outcome variable was coded as “1”. The outcome was based on the definitions and recommendations of the World Health Organization (WHO) regarding exclusively breastfeeding an infant for the first six months: “giving only breastfed milk, without any other food or liquid, even water” [45].

#### Independent variables

Covariables were identified from the literature review and the data available in the study [46,47]. They were (a) performance accomplishments: feeding only breastmilk before providing complementary foods to a previous child (yes/no), and not providing complementary foods (any food or liquid) or water to the previous child until 6 months of age (yes/no), (b) vicarious experience: observing other women breastfeeding, (c) verbal persuasion: father’s decision regarding breastfeeding (yes, no), living with parents in law (yes, no). We also adjusted the outcome variables by maternal age (<25, > = 25 years), maternal education (college or lower, university or higher), parity (primiparous, multiparous), and valued breastfeeding benefits (yes, no) in our multivariable analysis. Participants were asked the main reasons for breastfeeding their child with an open-ended question. If a mother selected one of seven options on benefits of breastfeeding including stating that breastmilk is the best baby food, helps the infant become smarter, prevent allergies, is cheaper, is the right thing to do, is more convenient for mothers, and is fashionable, she was coded as “yes: valued breastfeeding benefits”.

### Analysis

Descriptive statistics were used to summarize the sample characteristics and the frequency distribution of “breastfeeding intention” and “exclusive breastfeeding intention” to 6 months. Multivariable logistic regression analysis was performed to determine the factors associated with the two dependent variables (breastfeeding intention and exclusive breastfeeding intention to 6 months). Logistic regression models were fitted for three targeted groups (primiparous, multiparous, and all mothers). In these multivariable analyses, the included independent variables were maternal age, education level, seeing other women breastfeed, appreciating the benefits of breastfeeding, living with parents-in-law, the father’s decision regarding breastfeeding, and parity. In addition to those already being controlled, adjustments were further made by adding performance accomplishments variables (“feeding only breastmilk before providing complementary foods to a previous child”, and “not giving solid food and water to the previous child until 6 months of age”) in the logistic regression model for the multiparous group. Adjusted odds ratios (aOR) were calculated with 95% confidence intervals (95% CI) and reported. The goodness-of-fit of the models were assessed using the Hosmer—Lemeshow test. All statistical analyses were performed using SPSS (IBM Corp. Released 2017. IBM SPSS Statistics for Windows, Version 25.0. Armonk, NY: IBM Corp). Data is available in S1 Data.

### Ethical considerations

Interviewers introduced the study with written information and leaflets for all pregnant mothers visiting the antennal clinics. Mothers who agreed to participate in the study were asked to sign a written consent form before the interview. Participants understood that they could withdraw consent at any time without prejudice. Identities of individuals and establishments were kept strictly confidential. The study protocol was approved by the Curtin University Human Research Ethics Committee (Ref: HRE2019-0143-03) and the Ethical Review Board for Biomedical Research, Hanoi University of Public Health (Ref: 28/2019/YTCC-HD3). The trial registration number is ACTRN12619000531112.

## Results

### Participants’ characteristics

A total of 1230 pregnant women were included in the analysis of this study. The characteristics of pregnant women are shown in Table 1. Ages ranged from 18 to 45 years with a mean of 28.2 ± 4.7. Most of the pregnancies, 74.4%, were 25 years old or older. More than half, 56.3%, had obtained a university or higher level of education.

**Table 1:** Socio-demographic and relevant characteristics of the pregnant women (n = 1230).

Characteristics	n	%
Maternal age (years)		
< 25	315	25.6
> = 25	915	74.4
Education		
College or lower	538	43.7
University or higher	692	56.3
Seeing another woman breastfeed		
Yes	338	27.5
Valuing breastfeeding benefits		
Yes	532	43.3
Living with parents-in-law		
Yes	514	41.8
Father’s desire for his baby to be breastfed		
Yes	64	5.2
Parity		
Primiparous	609	49.5
Multiparous	621	50.5
Feeding the previous child with breastmilk only before complementary foods*		
Yes	360	58.0
Not giving solid foods or water to the previous child until 6 months of age*		
Yes	102	16.4
Intent to feed only breastmilk to 6 months		
Yes	737	59.9
Intent to breastfeed exclusive (without any solid foods and water) to 6 months		
Yes	513	41.7

* Among multiparous’ pregnant mothers, n = 621.

About 27.5% of pregnant women reported having seen other women breastfeed. The proportion of mothers who valued the benefits of breastfeeding was 43.3%, while only 5.2% of fathers preferred his baby to be breastfed. About 41.8% of participants were living with their parents-in-law. Nearly half of the participants (49.5%) were first-time mothers. About 59.9% of mothers had the intent to feed only breastmilk to 6 months. Intention to breastfeed exclusively (without any solid foods and water) to 6 months was reported by 41.7% of mothers.

### Factors associated with intention to breastfeed and exclusive breastfeeding

The factors associated with breastfeeding intention and exclusive breastfeeding intention among all mothers, both primiparous and multiparous are shown in Tables 2–4.

**Table 2:** Factors associated with breastfeeding intentions among mothers in Hanoi 2020 (n = 1230).

Factors	Breastfeeding intention*				Exclusive breastfeeding intention**			
	Yes, n (%)	No, n (%)	aOR (95% CI)	p	Yes, n (%)	No, n (%)	aOR (95%CI)	p
**Maternal age (years)**				**0.022**				**0.049**
< 25	140 (44.4)	175 (55.6)	Ref		202 (61.4)	113 (35.9)	Ref	
≥25	353 (38.6)	562 (61.4)	**1.41 (1.05–1.89)**		515 (56.3)	400 (43.7)	**1.35 (1.00–1.81)**	
**Education**				0.322				**0.009**
College or lower	236 (43.9)	302 (56.1)	Ref		348 (64.7)	190 (35.3)	Ref	
University or higher	257 (37.1)	435 (62.9)	1.13 (0.89–1.44)		369 (53.3)	323 (46.7)	**1.38 (1.08–1.76)**	
**Seeing another woman breastfeed**				0.052				**0.033**
No	357 (40.0)	535 (60.0)	Ref		527 (59.1)	365 (40.9)	Ref	
Yes	136 (40.2)	202 (59.8)	1.37 (0.99–1.92)		190 (56.2)	148 (43.8)	**1.43 (1.03–2.00)**	
**Valuing breastfeeding benefits**				0.202				0.068
No	225 (42.3)	307 (57.7)	Ref		326 (61.3)	206 (38.7)	Ref	
Yes	268 (38.4)	430 (61.6)	1.17 (0.92–1.48)		391 (56.0)	307 (44.0)	1.25 (0.98–1.58)	
**Living with parents in law**								**0.017**
Yes	226 (44.0)	288 (56.0)	Ref	0.071	327 (63.6)	187 (36.4)	Ref	
No	267 (37.3)	449 (62.7)	1.25 (0.98–1.59)		390 (54.5)	326 (45.5)	**1.34 (1.05–1.70)**	
**Father’s desire for his baby to be breastfed**				0.412				0.629
No	464 (39.8)	702 (60.2)	Ref		681 (58.4)	485 (41.6)	Ref	
Yes	29 (45.3)	35 (54.7)	1.24 (0.74–2.09)		36 (55.4)	28 (43.8)	0.88 (0.52–1.48)	
**Parity**				**<0.001**				**0.004**
Primiparous	221 (36.3)	388 (63.7)	Ref		342 (56.2)	267 (43.8)	Ref	
Multiparous	272 (43.8)	349 (56.2)	**1.85 (1.35–2.53)**		375 (60.4)	246 (39.6)	**1.60 (1.16–2.19)**	
			Hosmer and Lemeshow Test, p = 0.620				Hosmer and Lemeshow Test, p = 0.362	

*Intent to feed only breastmilk until 6 months.

** Intent to breastfeed exclusively (without any solid foods and water) until 6 months.

aOR (adjusted odds ratio) was adjusted with maternal age, education, seeing another woman breastfeed, valuing breastfeeding benefits, living with parents-in-law, the father’s desire for his baby to be breastfed, and parity.

**Table 3:** Factors associated with breastfeeding intentions among primiparous mothers in Hanoi 2020 (N = 609).

Factors	Breastfeeding intention				Exclusive breastfeeding intention			
	Yes, n (%)	No, n (%)	aOR, 95%CI	p	Yes, n (%)	No, n (%)	aOR, 95%CI	
**Maternal age (years)**				**0.003**				**0.002**
< 25	112 (44.8)	138 (55.2)	Ref		163 (65.2)	87 (34.8)	Ref	
≥25	109 (30.4)	250 (69.6)	**1.71 (1.20–2.44)**		179 (49.9)	180 (50.1)	**1.71 (1.21–2.42)**	
**Education**				0.213				0.439
College or lower	98 (41.9)	136 (58.1)	Ref		143 (61.1)	91 (38.9)	Ref	
University or higher	123 (32.8)	252 (67.2)	1.26 (0.88–1.80)		199 (53.1)	176 (46.9)	1.15 (0.81–1.63)	
**Valuing breastfeeding benefits**				0.509	171 (58.2)	123 (41.8)		0.447
No	114 (38.8)	180 (61.2)	Ref		171 (54.3)	144 (45.7)	Ref	
Yes	107 (34.0)	208 (66.0)	1.12 (0.80–1.58)				1.14 (0.82–1.58)	
**Living with parents in law**				0.292				0.109
Yes	109 (40.5)	160 (59.5)	Ref		166 (61.7)	103 (38.3)	Ref	
No	112 (32.9)	228 (67.1)	1.21 (0.85–1.71)		176 (51.8)	164 (48.2)	1.32 (0.94–1.85)	
**Father’s decision on breastfeeding**				**0.048**				0.913
No	202 (35.3)	370 (64.7)	Ref		321 (56.1)	251 (43.9)	Ref	
Yes	19 (51.4)	18 (48.6)	**2.01 (1.01–4.01)**		21 (56.8)	16 (43.2)	1.04 (0.52–2.08)	
			Hosmer and Lemeshow Test, p = 0.818				Hosmer and Lemeshow Test, p = 0.638	

*Intent to feed only breastmilk until 6 months.

** Intent to breastfeed exclusively (without any solid foods and water) until 6 months.

aOR (adjusted odds ratio) was adjusted with maternal age, education, valuing breastfeeding benefits, living with parents-in-law, father’s desire for his baby to be breastfed.

**Table 4:** Factors associated with breastfeeding intentions among multiparous mothers in Hanoi 2020 (N = 621).

Factors	Breastfeeding intention				Exclusive breastfeeding intention			
	Yes, n (%)	No, n (%)	aOR, 95%CI	p	Yes, n (%)	No, n (%)	aOR, 95%CI	p
**Maternal age (years)**				0.859				0.646
< 25	28 (43.1)	37 (56.9)	Ref		39 (60.0)	26 (40.0)	Ref	
≥ 25	244 (43.9)	312 (56.1)	0.95 (0.53–1.71)		336 (60.4)	220 (39.6)	0.87 (0.48–1.58)	
**Education**				0.800				**0.003**
College or lower	138 (45.4)	166 (54.6)	Ref		205 (67.4)	99 (32.6)	Ref	
University or higher	134 (42.3)	188 (57.7)	1.05 (0.73–1.50)		170 (53.6)	151 (46.7)	**1.74 (1.21–2.51)**	
**Seeing another woman breastfeed**				0.057				0.089
No	136 (48.1)	147 (51.9)	Ref		185 (65.4)	98 (34.6)	Ref	
Yes	136 (40.2)	202 (59.8)	1.40 (0.99–1.99)		190 (56.2)	148 (43.8)	1.36 (0.95–1.94)	
**Valuing breastfeeding benefits**				0.274				0.156
No	111 (46.6)	127 (53.4)	Ref		83 (34.9)	155 (65.1)	Ref	
Yes	222 (58.0)	161 (42.0)	1.22 (0.85–1.75)		163 (42.6)	220 (57.4)	1.30 (0.90–1.88)	
**Living with parents in law**				0.096				0.147
Yes	117 (47.8)	128 (52.2)	Ref		161 (65.7)	84 (34.3)	Ref	
No	155 (41.2)	221 (58.8)	1.36 (0.95–1.95)		214 (56.9)	162 (43.1)	1.31 (0.91–1.90)	
**Father’s desire for his baby to be breastfed**				0.216				0.190
No	262 (44.1)	332 (55.9)	Ref		360 (60.6)	234 (39.4)	Ref	
Yes	10 (37.0)	17 (63.0)	0.57 (0.24–1.38)		15 (55.6)	12 (44.4)	0.57 (0.25–1.32)	
**Feeding the previous child with breastmilk only before complementary foods**				**<0.001**				**<0.001**
No	169 (64.8)	92 (35.2)	Ref		67 (25.7)	194 (74.3)	Ref	
Yes	103 (28.6)	257 (71.4)	**4.64 (3.28–6.57)**		179 (49.7)	181 (50.3)	**2.90 (2.02–4.17)**	
**Not giving solid foods or water to the previous child until 6 months of age**				0.074				
No	280 (53.9)	239 (46.1)	Ref		177 (34.1)	342 (65.9)	Ref	**<0.001**
Yes	69 (67.6)	33 (32.4)	1.56 (0.96–2.56)		69 (67.6)	33 (32.4)	**4.86 (2.38–6.27)**	
			Hosmer and Lemeshow Test, p = 0.199				Hosmer and Lemeshow Test, p = 0.369	

*Intent to feed only breastmilk until 6 months.

** Intent to breastfeed exclusively (without any solid foods and water) until 6 months.

aOR (adjusted odds ratio) was adjusted with maternal age, education, seeing another woman breastfeed, valuing breastfeeding benefits, living with parents-in-law, the father’s desire for his baby to be breastfed, feeding the previous child with breastmilk only before complementary foods, and not giving solid foods or water to the previous child until 6 months of age.

Among all mothers (Table 2), the intention to breastfeed was associated with the mother’s age and parity. Mothers who were 25 years old or older were more likely to intend to breastfeed their newborn (aOR = 1.41, 95%CI: 1.05–1.89). Multiparous mothers had higher odds of breastfeeding intention than primiparous mothers (aOR = 1.85, 95%CI: 1.35–2.53). Higher odds of intention to breastfeed exclusively were found among mothers who were 25 years or older (aOR = 1.35, 95%CI: 1.00–1.81), had a university or higher level of education (aOR = 1.38, 95% CI: 1.08–1.76), were not living with parents-in-law (aOR = 1.34, 95% CI:1.05–1.70), observed another woman breastfeeding (aOR = 1.43, 95%CI:1.03–2.00), and were multiparous (aOR = 1.60, 95% CI:1.16–2.19).

As indicated in Table 3, primiparous mothers were more likely to intend to breastfeed (aOR = 1.71, 95%CI: 1.20–2.44) or exclusively breastfeed for up to 6 months (aOR = 1.71, 95%CI: 1.21–2.42) if they were 25 years of age or older. The father’s support for breastfeeding a child was positively associated with breastfeeding intention among first-time mothers (aOR = 2.01, 95%CI: 1.01–4.01).

Multiparous mothers were more likely to intend to breastfeed if they had fed the previous child only with breastmilk before complementary foods were introduced (aOR = 4.64, 95%CI: 3.28–6.57, Table 4). Intention to breastfeed exclusively among multiparous mothers was positively associated with the mother’s education at the undergraduate level or higher (aOR = 1.74, 95%CI: 1.21–2.51), feeding the previous child with breastmilk only before complementary foods (aOR = 2.90, 95%CI: 2.02–4.17), and not giving complementary foods to the previous child before 6 months of age (aOR = 4.86, 95%CI: 2.38–6.27).

## Discussion

The study aimed to identify the factors associated with breastfeeding intentions (“breastfeeding intention” and “exclusive breastfeeding intention” to 6 months) among pregnant mothers in Hanoi, Vietnam. Antenatal breastfeeding intention is a strong indicator of breastfeeding outcomes [13–17]. We found 59.9% of women intended to breastfeed and 41.7% to breastfeed exclusively to 6 months in Hanoi, Vietnam. These rates are much lower compared with data from a previous study in the city of Da Nang (74%) [48]. One possible explanation for the difference is that our study was among pregnant women while the previous study was conducted among postpartum women for whom the Da Nang Hospital had implemented the Essential Newborn Care package which included establishing skin-to-skin contact as soon as possible and breastfeeding within one hour after delivery [48]. This difference suggests that women may need additional support during the early postpartum and breastfeeding periods to practice breastfeeding regardless off having prenatal breastfeeding intentions [49]. Qualitative studies using focus groups may provide more insight into barriers to breastfeeding intention among pregnant women.

Across all mothers (including both primiparous and multiparous), older maternal age, higher educational level, seeing another woman breastfeeding, not living with parents-in-law, and being multiparous mothers were positively associated with exclusive breastfeeding intention to 6 months. In the available literature, there appears to be no consistent pattern in breastfeeding intention based on age groups. While some previous studies have suggested that mothers of an older age are more likely to have breastfeeding intention [14], others suggest that younger mothers do [20,27]. In our study, we found mothers of 25 years or older had a higher intent to breastfeed. This aligns with findings that the higher mother’s age, the better knowledge and confidence in breastfeeding she may possess [50].

We found that a higher maternal educational level (university or higher), was positively associated with intent to breastfeed exclusively. This finding was similar to previous studies in different countries [19,20,27]. Higher education could help mothers be fully aware of the benefits of exclusive breastfeeding without any other liquids or solid food before 6 months of age. Women who intended to breastfeed possessed a greater knowledge about prenatal nutritional recommendations and accessed more sources of information about prenatal and infant nutrition compared with their non-intending counterparts [18].

Seeing other women breastfeeding may positively influence maternal attitudes towards breastfeeding, breastfeeding intentions, and infant feeding decisions [22,23]. However, the proportion of mothers who saw other women breastfeeding was relatively low at 27.5% in our study and only two were first-time mothers (data not shown). A previous study in rural areas in Vietnam found that only 36.1% of mothers reporting felt comfortable with breastfeeding in public places [43]. Normalizing breastfeeding practice should be a focus in designing communication campaigns to improve breastfeeding outcomes.

Not living with parents–in–law was also a positive predictor of intention to breastfeed exclusively for six months across all mothers. In our study, nearly half ofpregnant women were living with their parents-in-law. Previous studies have reported that mothers-in-law often help with cooking, housework, and taking care of a child when mothers return to work [51,52]. Mothers-in-law have a strong influence on infant feeding practices. Feeding a newborn with formula milk is often perceived as a way for family members to support mothers recovering after birth by [53,54].

Previous literature on the intention to breastfeed have indicated that that parity and previous breastfeeding experience are important factors [15,19–21]. The present study is the first analysis that has been undertaken separately for primiparous and multiparous women in Vietnam. High parity is a positive predicting factor of breastfeeding intentions, similar to results from previous studies on breastfeeding outcomes in other countries [55–57].

Among first-time mothers with no experience in breastfeeding, the husband’s opinion is also a factor influencing mother’s breastfeeding intention. In Vietnam, the involvement of fathers in breastfeeding promotion programs could increase the early breastfeeding initiation rate, as well as exclusive breastfeeding at 4 and 6 months [44,58]. In other countries, women with supportive partners or sharing parenting support were more likely to intend to breastfeed [19,20,22,24,25,59].

Among multiparous women, good breastfeeding practices with their previous child, or children, was a positive factor associated with breastfeeding intentions for the following children. A 2018 scoping review in South Asia suggested that to support optimal breastfeeding, programs and interventions should reach women and their families repeatedly beginning in early pregnancy [60]. Optimal breastfeeding support for women resulting in good experiences with breastfeeding, particularly after delivery and before discharge from the hospital could thus encourage women to continue breastfeeding [49].

There are some limitations to be considered when interpreting the results of this study. Mothers who were involved in the study were recruited from district- and city-level hospitals that may cater to a higher social–economic status than other hospitals, thus, recruited mothers may have had a higher educational level or levels of knowledge on breastfeeding. Subjects who agreed to participate in the study may also have paid more attention to maternal and child health care in general and in breastfeeding in particular. The breastfeeding intentions identified in this study, therefore, could have been overestimated. In addition, some identified associated factors from the previous studies were not included in the analysis, fsuch as, health conditions during pregnancy [19], household economics [20,26], receiving counseling from health professionals [26,27,61], and breastfeeding self‐efficacy [15,62]. To minimize bias in sample selection within the hospitals, consecutive sampling was used in this study.

Despite these limitations, we believe the two selected hospitals are generally representative of urban areas in Vietnam. Dong Anh General District Hospital is one of 17 semi-urban districts in Hanoi and has 6,000 deliveries per year. The Hanoi Obstetrics and Gynecology Hospital is a specialist hospital in obstetrics and gynecology in the northern region and mainly provides services to people living in urban with about 44,000 deliveries annually. The survey also applied a standard and validated questionnaire which minimized response bias. Separately analyzing resultsfor primiparous and multiparous women was also helpful for identifying relevant breastfeeding consultations and interventions. The results of this study could be supplemented by further follow-up studies in different areas of Vietnam to gain a better understanding of breastfeeding intentions and their associated factors and actual outcomes.

## Conclusions

This is the first analysis reporting separately for primiparous and multiparous women on factors associated with breastfeeding intention and exclusive breastfeeding intention to six months in Vietnam. The study found that husband’s desire for the infant to be breastfed was associated with breastfeeding intention among first-time mothers while good breastfeeding practices (breastfeeding as predominant foods, not giving any solid foods or water until 6 months) with the previous child were the associated with breastfeeding intentions among multiparous women. The results suggested that inexperienced mothers (first-time mothers and mothers without exclusive breastfeeding practices for the previous child) should be provided with greater support to promote exclusive breastfeeding.

## Supporting information

S1 Table Socio-demographic and relevant characteristics of the pregnant women (n = 1230). (DOCX)

S2 Table Factors associated with breastfeeding intention among mothers in Hanoi 2020 (n = 1230). (DOCX)

S3 Table Factors associated with breastfeeding intention among primiparous mothers in Hanoi 2020 (N = 609). (DOCX)

S4 Table Factors associated with breastfeeding intention among multiparous mothers in Hanoi 2020 (N = 621). (DOCX)

S1 File Questionnaire in Vietnamese. (DOCX)

S2 File Questionnaire in English. (DOCX)

S1 Data (SAV)
