# Supplementary material for: Factors associated with intention to breastfeed in Vietnamese mothers: A cross-sectional study
Source: PLoS One. 2023 Dec 12;18(12):e0279691. doi: 10.1371/journal.pone.0279691 (PMC10715656; doi:10.1371/journal.pone.0279691)
Supplement: S1 Table — (DOCX) [file pone.0279691.s001.docx]

### Table 1 Socio-demographic and relevant characteristics of the pregnant women (n=1230)

| Characteristics | n | % |
| --- | --- | --- |
| Maternal age (years) |  |  |
| < 25 | 315 | 25.6 |
| >=25 | 915 | 74.4 |
| Education |  |  |
| College or lower | 538 | 43.7 |
| University or higher | 692 | 56.3 |
| Seeing another woman breastfeed |  |  |
| Yes | 338 | 27.5 |
| Valuing breastfeeding benefits |  |  |
| Yes | 532 | 43.3 |
| Living with parents-in-law |  |  |
| Yes | 514 | 41.8 |
| Father’s desire for his baby to be breastfed |  |  |
| Yes | 64 | 5.2 |
| Parity |  |  |
| Primiparous | 609 | 49.5 |
| Multiparous | 621 | 50.5 |
| Feeding the previous child with breastmilk only before complementary foods* |  |  |
| Yes | 360 | 58.0 |
| Not giving solid foods or water to the previous child until 6 months of age* |  |  |
| Yes | 102 | 16.4 |
| Intent to feed only breastmilk to 6 months |  |  |
| Yes | 737 | 59.9 |
| Intent to exclusive breastfeeding (without any solid foods and water) to 6 months |  |  |
| Yes | 513 | 41.7 |

* Among multiparous’ pregnant mothers, n = 621
