# Supplementary material for: Factors associated with intention to breastfeed in Vietnamese mothers: A cross-sectional study
Source: PLoS One. 2023 Dec 12;18(12):e0279691. doi: 10.1371/journal.pone.0279691 (PMC10715656; doi:10.1371/journal.pone.0279691)
Supplement: S2 Table — (DOCX) [file pone.0279691.s002.docx]

### Table 2 Factors associated with breastfeeding intention among mothers in Hanoi 2020 (n=1230)

| **Characteristics** | **Breastfeeding intention*** | | | | **Exclusive breastfeeding intention**** | | | |
| --- | --- | --- | --- | --- | --- | --- | --- | --- |
|  | Yes, n (%) | No, n (%) | aOR (95% CI) | p | Yes, n (%) | No, n (%) | aOR (95%CI) | p |
| **Maternal age (years)** |  |  |  | **0.02**2 |  |  |  | **0.049** |
| < 25 | 140 (44.4) | 175 (55.6) | Ref |  | 202 (61.4) | 113 (35.9) | Ref |  |
| ≥25 | 353 (38.6) | 562 (61.4) | **1.41 (1.05 - 1.89)** |  | 515 (56.3) | 400 (43.7) | **1.35 (1.00 - 1.81)** |  |
| **Education** |  |  |  | 0.322 |  |  |  | **0.009** |
| College or lower | 236 (43.9) | 302 (56.1) | Ref |  | 348 (64.7) | 190 (35.3) | Ref |  |
| University or higher | 257 (37.1) | 435 (62.9) | 1.13 (0.89 - 1.44) |  | 369 (53.3) | 323 (46.7) | **1.38 (1.08 - 1.76)** |  |
| **Seeing another woman breastfeed** |  |  |  | 0.052 |  |  |  | **0.033** |
| No | 357 (40.0) | 535 (60.0) | Ref |  | 527 (59.1) | 365 (40.9) | Ref |  |
| Yes | 136 (40.2) | 202 (59.8) | 1.37 (0.99 - 1.92) |  | 190 (56.2) | 148 (43.8) | **1.43 (1.03 - 2.00)** |  |
| **Valuing breastfeeding benefits** |  |  |  | 0.202 |  |  |  | 0.068 |
| No | 225 (42.3) | 307 (57.7) | Ref |  | 326 (61.3) | 206 (38.7) | Ref |  |
| Yes | 268 (38.4) | 430 (61.6) | 1.17 (0.92 - 1.48) |  | 391 (56.0) | 307 (44.0) | 1.25 (0.98 - 1.58) |  |
| **Living with parents in law** |  |  |  |  |  |  |  | **0.017** |
| Yes | 226 (44.0) | 288 (56.0) | Ref | 0.071 | 327 (63.6) | 187 (36.4) | Ref |  |
| No | 267 (37.3) | 449 (62.7) | 1.25 (0.98 - 1.59) |  | 390 (54.5) | 326 (45.5) | **1.34 (1.05 - 1.70)** |  |
| **Father’s desire for his baby to be breastfed** |  |  |  | 0.412 |  |  |  | 0.629 |
| No | 464 (39.8) | 702 (60.2) | Ref |  | 681 (58.4) | 485 (41.6) | Ref |  |
| Yes | 29 (45.3) | 35 (54.7) | 1.24 (0.74 - 2.09) |  | 36 (55.4) | 28 (43.8) | 0.88 (0.52 - 1.48) |  |
| **Parity** |  |  |  | **<0.001** |  |  |  | **0.004** |
| Primiparous | 221 (36.3) | 388 (63.7) | Ref |  | 342 (56.2) | 267 (43.8) | Ref |  |
| Multiparous | 272 (43.8) | 349 (56.2) | **1.85 (1.35 - 2.53)** |  | 375 (60.4) | 246 (39.6) | **1.60 (1.16 - 2.19)** |  |
|  |  |  | Hosmer and Lemeshow Test, p=0.620 | |  |  | Hosmer and Lemeshow Test, p=0.362 | |
| *Intent to feed only breastmilk until 6 months  ** Intent to exclusive breastfeeding (without any solid foods and water) until 6 months | | | | | | | | |
