# Supplementary material for: Factors associated with intention to breastfeed in Vietnamese mothers: A cross-sectional study
Source: PLoS One. 2023 Dec 12;18(12):e0279691. doi: 10.1371/journal.pone.0279691 (PMC10715656; doi:10.1371/journal.pone.0279691)
Supplement: S3 Table — (DOCX) [file pone.0279691.s003.docx]

### Table 3 Factors associated with breastfeeding intention among primiparous mothers in Hanoi 2020 (N=609)

| **Characteristics** | **Breastfeeding intention** | | | | **Exclusive breastfeeding intention** | | | |
| --- | --- | --- | --- | --- | --- | --- | --- | --- |
|  | Yes, n (%) | No, n (%) | aOR, 95%CI | p | Yes, n (%) | No, n (%) | aOR, 95%CI |  |
| **Maternal age (years)** |  |  |  | **0.003** |  |  |  | **0.002** |
| < 25 | 112 (44.8) | 138 (55.2) | Ref |  | 163 (65.2) | 87 (34.8) | Ref |  |
| ≥25 | 109 (30.4) | 250 (69.6) | **1.71 (1.20 - 2.44)** |  | 179 (49.9) | 180 (50.1) | **1.71 (1.21 - 2.42)** |  |
| **Education** |  |  |  | 0.213 |  |  |  | 0.439 |
| College or lower | 98 (41.9) | 136 (58.1) | Ref |  | 143 (61.1) | 91 (38.9) | Ref |  |
| University or higher | 123 (32.8) | 252 (67.2) | 1.26 (0.88 - 1.80) |  | 199 (53.1) | 176 (46.9) | 1.15 (0.81 - 1.63) |  |
| **Valuingbreastfeeding benefits** |  |  |  | 0.509 | 171 (58.2) | 123 (41.8) |  | 0.447 |
| No | 114 (38.8) | 180 (61.2) | Ref |  | 171 (54.3) | 144 (45.7) | Ref |  |
| Yes | 107 (34.0) | 208 (66.0) | 1.12 (0.80 - 1.58) |  |  |  | 1.14 (0.82 - 1.58) |  |
| **Living with parents in law** |  |  |  | 0.292 |  |  |  | 0.109 |
| Yes | 109 (40.5) | 160 (59.5) | Ref |  | 166 (61.7) | 103 (38.3) | Ref |  |
| No | 112 (32.9) | 228 (67.1) | 1.21 (0.85 - 1.71) |  | 176 (51.8) | 164 (48.2) | 1.32 (0.94 - 1.85) |  |
| **Father’s decision on breastfeeding** |  |  |  | **0.048** |  |  |  | 0.913 |
| No | 202 (35.3) | 370 (64.7) | Ref |  | 321 (56.1) | 251 (43.9) | Ref |  |
| Yes | 19 (51.4) | 18 (48.6) | **2.01 (1.01 - 4.01)** |  | 21 (56.8) | 16 (43.2) | 1.04 (0.52 - 2.08) |  |
|  |  |  | Hosmer and Lemeshow Test, p=0.818 | |  |  | Hosmer and Lemeshow Test, p=0.638 | |
| *Intent to feed only breastmilk until 6 months  ** Intent to exclusive breastfeeding (without any solid foods and water) until 6 months | | | | | | | | |
