# Supplementary material for: Factors associated with intention to breastfeed in Vietnamese mothers: A cross-sectional study
Source: PLoS One. 2023 Dec 12;18(12):e0279691. doi: 10.1371/journal.pone.0279691 (PMC10715656; doi:10.1371/journal.pone.0279691)
Supplement: S4 Table — (DOCX) [file pone.0279691.s004.docx]

### Table 4 Factors associated with breastfeeding intention among multiparous mothers in Hanoi 2020 (N=621)

| **Characteristics** | **Breastfeeding intention** | | | | **Exclusive breastfeeding intention** | | | |
| --- | --- | --- | --- | --- | --- | --- | --- | --- |
|  | Yes, n (%) | No, n (%) | aOR, 95%CI | p | Yes, n (%) | No, n (%) | aOR, 95%CI | p |
| **Maternal age (years)** |  |  |  | 0.859 |  |  |  | 0.646 |
| < 25 | 28 (43.1) | 37 (56.9) | Ref |  | 39 (60.0) | 26 (40.0) | Ref |  |
| ≥ 25 | 244 (43.9) | 312 (56.1) | 0.95 (0.53 - 1.71) |  | 336 (60.4) | 220 (39.6) | 0.87 (0.48 - 1.58) |  |
| **Education** |  |  |  | 0.800 |  |  |  | **0.003** |
| College or lower | 138 (45.4) | 166 (54.6) | Ref |  | 205 (67.4) | 99 (32.6) | Ref |  |
| University or higher | 134 (42.3) | 188 (57.7) | 1.05 (0.73 - 1.50) |  | 170 (53.6) | 151 (46.7) | **1.74 (1.21 - 2.51)** |  |
| **Seeing another woman breastfeed** |  |  |  | 0.057 |  |  |  | 0.089 |
| No | 136 (48.1) | 147 (51.9) | Ref |  | 185 (65.4) | 98 (34.6) | Ref |  |
| Yes | 136 (40.2) | 202 (59.8) | 1.40 (0.99 - 1.99) |  | 190 (56.2) | 148 (43.8) | 1.36 (0.95 - 1.94) |  |
| **Valuing breastfeeding benefits** |  |  |  | 0.274 |  |  |  | 0.156 |
| No | 111 (46.6) | 127 (53.4) | Ref |  | 83 (34.9) | 155 (65.1) | Ref |  |
| Yes | 222 (58.0) | 161 (42.0) | 1.22 (0.85 - 1.75) |  | 163 (42.6) | 220 (57.4) | 1.30 (0.90 - 1.88) |  |
| **Living with parents in law** |  |  |  | 0.096 |  |  |  | 0.147 |
| Yes | 117 (47.8) | 128 (52.2) | Ref |  | 161 (65.7) | 84 (34.3) | Ref |  |
| No | 155 (41.2) | 221 (58.8) | 1.36 (0.95 - 1.95) |  | 214 (56.9) | 162 (43.1) | 1.31 (0.91 - 1.90) |  |
| **Father’s desire for his baby to be breastfed** |  |  |  | 0.216 |  |  |  | 0.190 |
| No | 262 (44.1) | 332 (55.9) | Ref |  | 360 (60.6) | 234 (39.4) | Ref |  |
| Yes | 10 (37.0) | 17 (63.0) | 0.57 (0.24 - 1.38) |  | 15 (55.6) | 12 (44.4) | 0.57 (0.25 - 1.32) |  |
| **Feeding the previous child with breastmilk only before complementary foods** |  |  |  | **<0.001** |  |  |  | **<0.001** |
| No | 169 (64.8) | 92 (35.2) | Ref |  | 67 (25.7) | 194 (74.3) | Ref |  |
| Yes | 103 (28.6) | 257 (71.4) | **4.64 (3.28 - 6.57)** |  | 179 (49.7) | 181 (50.3) | **2.90 (2.02 - 4.17)** |  |
| **Not giving solid foods or water to the previous child until 6 months of age** |  |  |  | 0.074 |  |  |  |  |
| No | 280 (53.9) | 239 (46.1) | Ref |  | 177 (34.1) | 342 (65.9) | Ref | **<0.001** |
| Yes | 69 (67.6) | 33 (32.4) | 1.56 (0.96 - 2.56) |  | 69 (67.6) | 33 (32.4) | **4.86 (2.38 - 6.27)** |  |
|  |  |  | Hosmer and Lemeshow Test, p=0.199 | |  |  | Hosmer and Lemeshow Test, p=0.369 | |
| *Intent to feed only breastmilk until 6 months  ** Intent to exclusive breastfeeding (without any solid foods and water) until 6 months | | | | | | | | |
