# Supplementary material for: Factors associated with intention to breastfeed in Vietnamese mothers: A cross-sectional study
Source: PLoS One. 2023 Dec 12;18(12):e0279691. doi: 10.1371/journal.pone.0279691 (PMC10715656; doi:10.1371/journal.pone.0279691)
Supplement: S1 File — (DOCX) [file pone.0279691.s005.docx]

**Nghiên cứu chăm sóc sức khỏe bà mẹ trẻ em**

**Bộ câu hỏi baseline**

**ID___________________________**

| **Câu hỏi** | **Trả lời** |
| --- | --- |
| 1. Chị bao nhiêu tuổi |  |
| 2. Trình độ học vấn cao nhất của chị trước khi mang thai? | 1. Tiểu học 2. Trung học cơ sở 3. Trung học 4. Trung cấp/cao đẳng 5. Đại học/Sau đại học |
| 3. Hiện chị đang sống cùng ai? | 1. Chồng; 2. Bố mẹ đẻ; 3. Bố mẹ chồng;  4. Khác (ghi rõ) |
| 4. Lần sinh này là lần sinh thứ mấy của chị? (không tính thai lưu) | …..  Nếu sinh lần 1, chuyển sang câu 7 |
| **Câu hỏi về lần sinh gần nhất** | |
| 5. Chị bắt đầu cho bé ăn sam, ăn dặm khi cháu được bao nhiêu tháng tuổi? | ……………………………………………… |
| 6. Trước khi cho trẻ ăn sam, ăn dặm, thức ăn của trẻ là? | 1. Chỉ có sữa mẹ 2. Sữa mẹ và sữa công thức  3. Sữa công thức 4. Khác (ghi rõ) |
| **Câu hỏi cho lần sinh sắp tới** | |
| 7. Người thân, họ hàng của chị có cho trẻ bú mẹ không? | 1. Không  2. Có |
| 8. Chị dự định sẽ nuôi con bằng sữa mẹ, sữa công thức hay cả hai? | 1. Chỉ có sữa mẹ 2. Sữa mẹ và sữa công thức  3. Sữa công thức 4. Không biết 5. Khác (ghi rõ) |
| 9. Khi nào chị định cho con uống thêm các loại nước khác như nước trắng, nước hoa quả? | 1. <3 tháng 2. Ba tháng 3. Bốn tháng 4. Năm tháng 5. Sáu tháng 6. Sau sáu tháng |
| 10. Khi nào chị định cho trẻ ăn dặm/ăn bổ sung thức ăn? | 1. <3 tháng 2. Ba tháng 3. Bốn tháng 4. Năm tháng 5. Sáu tháng 6. Sau sáu tháng |

11. Theo chị lý do phụ nữ cho trẻ bú mẹ vì? (Đọc to hết các đáp án)

|  | Không…1 | Có…2 |
| --- | --- | --- |
| 1.     Bố trẻ muốn trẻ được bú mẹ |  |  |
| 2.     Sữa mẹ tốt hơn cho trẻ nhỏ |  |  |
| 3.     Cho trẻ bú mẹ là điều đúng nên làm |  |  |
| 4.     Cho trẻ bú mẹ rẻ hơn |  |  |
| 5.   Trẻ được bú mẹ thông minh hơn |  |  |
| 6.   Cho trẻ bú mẹ giúp mẹ giảm cân |  |  |
| 7.     Cho trẻ bú mẹ là hợp thời |  |  |
| 8.     Mẹ/mẹ chồng khuyên tôi cho trẻ bú mẹ |  |  |
| 9.     Người khác khuyên tôi cho trẻ bú mẹ |  |  |
| 10.  Cho bú mẹ giúp trẻ phòng được dị ứng |  |  |
| 11.  Cho trẻ bú mẹ thuận lợi hơn |  |  |
| 12.  Khác (Ghi rõ) | | |
