# Supplementary material for: Factors associated with intention to breastfeed in Vietnamese mothers: A cross-sectional study
Source: PLoS One. 2023 Dec 12;18(12):e0279691. doi: 10.1371/journal.pone.0279691 (PMC10715656; doi:10.1371/journal.pone.0279691)
Supplement: S2 File — (DOCX) [file pone.0279691.s006.docx]

**Vietnam Infant Feeding Study**

**Record ID___________________________**

| **Questions** | **Answers** |
| --- | --- |
| 1. How old are you (years)? |  |
| 2. What is your highest level of education completed? (pregnant woman) | 1. Primary school 2. Secondary school 3. High school 4. College 5. University/Post-graduate |
| 3. Who are you now living with? | 1. Husband; 2. Parents; 3. Parents in law;  4. Others (please specify) |
| 4. How many children have you given birth, excluding stillbirth? | …..  If 1, move to Question 7 |
| The following questions are on your last baby |  |
| 5. How old was your baby when he or she was first fed with foods or drink? | …………………………………………… |
| 6. Before complementary feeding, what were the main foods? | 1. Breastmilk 2. Some formula + breastmilk; 3. Formula milk; 4. Other (specify) |
| The following questions are on your coming baby |  |
| 7. Have you watched any of your close relatives breastfeed their babies? | 1. No  2. Yes |
| 8. How do you intend to feed your infant? | 1. Breastmilk only from birth 2. Some formula + breastmilk; 3. Formula milk only; 4. Don’t know;  5. Other (specify______________________) |
| 9. When do you expect to give your infant solid foods? | 1. <3 months 2. Three months 3. Four months 4. Five months 5. Six months 6. More than six months |
| 10. When do you expect to give your infant water to drink? | 1. <3 months 2. Three months 3. Four months 4. Five months 5. Six months 6. More than six months |

11. How were each of the following reasons for your decision to breastfeed your baby? Readout loud all options

|  | NO…1 | YES…2 |
| --- | --- | --- |
| 1.     The baby’s father wanted me to breastfeed |  |  |
| 2.     Breastmilk is better for the baby |  |  |
| 3.     Breastfeeding is the right thing to do |  |  |
| 4.     Breastfeeding is cheaper |  |  |
| 5.     Breastfed babies are more intelligent |  |  |
| 6.     Breastfeeding helps me lose weight |  |  |
| 7.     Breastfeeding is fashionable |  |  |
| 8.     My mother/mother-in-law advised me to breastfeed |  |  |
| 9.     Other people advised me to breastfeed |  |  |
| 10.  Breastfeeding helps prevent allergies |  |  |
| 11.  Breastfeeding is more convenient |  |  |
| 12.  Other (please specify) | | |
